# Natural compounds as potential adjuvants to cancer therapy: Preclinical evidence

**DOI:** 10.1111/bph.14816

**Published:** 2019-11-27

**Authors:** Shian‐Ren Lin, Chia‐Hsiang Chang, Che‐Fang Hsu, May‐Jwan Tsai, Henrich Cheng, Max K. Leong, Ping‐Jyun Sung, Jian‐Chyi Chen, Ching‐Feng Weng

**Affiliations:** ^1^ Department of Life Science and Institute of Biotechnology National Dong Hwa University Hualien Taiwan; ^2^ Center for Prevention and Therapy of Gynaecological Cancers, Department of Research Tzu Chi Hospital Hualien Taiwan; ^3^ Neural Regeneration Laboratory, Neurological Institute Taipei Veterans General Hospital Taipei City Taiwan; ^4^ Department of Chemistry National Dong Hwa University Hualien Taiwan; ^5^ Graduate Institute of Marine Biotechnology National Dong Hwa University Pingtung Taiwan; ^6^ Department of Biotechnology Southern Taiwan University of Science and Technology Tainan City Taiwan; ^7^ Department of Basic Medical Science, Center for Transitional Medicine Xiamen Medical College Xiamen China

## Abstract

Traditional chemotherapy is being considered due to hindrances caused by systemic toxicity. Currently, the administration of multiple chemotherapeutic drugs with different biochemical/molecular targets, known as combination chemotherapy, has attained numerous benefits like efficacy enhancement and amelioration of adverse effects that has been broadly applied to various cancer types. Additionally, seeking natural‐based alternatives with less toxicity has become more important. Experimental evidence suggests that herbal extracts such as *Solanum nigrum* and *Claviceps purpurea* and isolated herbal compounds (e.g., curcumin, resveratrol, and matairesinol) combined with antitumoral drugs have the potential to attenuate resistance against cancer therapy and to exert chemoprotective actions. Plant products are not free of risks: Herb adverse effects, including herb–drug interactions, should be carefully considered.

**Linked Articles:**

This article is part of a themed section on The Pharmacology of Nutraceuticals. To view the other articles in this section visit http://onlinelibrary.wiley.com/doi/10.1111/bph.v177.6/issuetoc

**Abbreviations**
EGCG: http://www.guidetopharmacology.org/GRAC/LigandDisplayForward?ligandId=7002
EMT: epithelial‐mesenchymal transition
MRP: http://www.guidetopharmacology.org/GRAC/FamilyDisplayForward?familyId=153
TCM: traditional Chinese medicine
NSCLC: non‐small‐cell lung cancer
OSCC: oral squamous cell carcinoma

## 1 INTRODUCTION

In clinics, chemotherapy for cancer patients is commonly based on the drug indications, recommended dosages, treatment duration, and adverse effects (e.g., nephrotoxicity and hepatotoxicity; Grossi et al., 2010; Sharbaf, Farhangi, & Assadi, 2017; Sulthana et al., 2017). Occasionally, it is difficult to prevent occurrences of adverse effects from chemotherapeutic drugs during therapy. For instance, http://www.guidetopharmacology.org/GRAC/LigandDisplayForward?ligandId=7069, a widely used chemotherapy agent, frequently induces cardiomyopathy and chronic heart failure with a prevalence between 4% and 36% (cardiomyopathy) and 0.2–8.7% (chronic heart failure) according to cumulative doses (Chatterjee, Zhang, Honbo, & Karliner, 2010; Volkova & Russell, 2011; J. Yu et al., 2018). Although there are reports of nephrotoxicity and immunosuppression from http://www.guidetopharmacology.org/GRAC/LigandDisplayForward?ligandId=5343 during treatment, it is, nevertheless, a first choice for the treatment of advanced non‐small‐cell lung cancer cells (NSCLC), breast cancer and ovarian cancer (Brown et al., 2013; Browning et al., 2017; Lorusso, Petrelli, Coinu, Raspagliesi, & Barni, 2014; Manohar & Leung, 2018). Moreover, cancer cells may develop drug resistance during treatment with chemotherapy. Accordingly, higher doses need to be applied to achieve a similar tumoricidal effect as the initial dosage. Frequently, higher dosages have a higher possibility of severe side effects (Zheng, 2017). Consequently, taking a combination of drugs with different mechanisms could synergistically potentiate therapeutic efficacy (Glasgow & Chougule, 2015).

Currently, combinations of chemotherapeutic drugs are widely used for various cancer types (Liu et al., 2017; Zhang et al., 2011). Importantly, the advantage of using multiple drugs are seen as the lowering of doses which could lead to lower resistance and the retention of the same efficacy or sometimes a higher efficacy, a synergistic effect (Glasgow & Chougule, 2015; He et al., 2015; Liboiron & Mayer, 2014). The effects of lower toxicity are ignored as they are considered to be harmless. In fact, the accumulation of toxicity from each drug can still cause deleterious systemic responses (F. Li & Zhang, 2015). Therefore, optimizing drug ratios and schedules can provide an opportunity to improve drug combination activity and reduce dosages to attenuate toxicity (L. Wu, Leng, Cun, Foged, & Yang, 2017). Additionally, drug combinations which include dietary supplements and natural products have been postulated to obtain similar effects to conventional chemotherapeutic drugs but with less adverse effects (Lin, Fu, Tsai, Cheng, & Weng, 2017). Three meta‐analyses reviewing traditional herbal medicine have found such products used as chemotherapeutic adjuvants in nasopharyngeal, breast, and pancreatic cancer treatments. The effective outcome has exemplified traditional herbal medicine as a chemotherapeutic adjuvant (W. Kim, Lee, Lee, Min, Baek, et al., 2015a; W. Kim, Lee, Lee, Min, Lee, & Cho, 2015b; Kuo et al., 2018). Since 2006, several clinical trials were conducted to assess the enhancing effect of natural compounds such as http://www.guidetopharmacology.org/GRAC/LigandDisplayForward?ligandId=7000 or traditional Chinese medicine (TCM) in promoting conventional chemotherapy against various cancers, including lung cancer (especially non‐small‐cell lung cancer), breast cancer, and colon cancer (Table 1). The conventional chemotherapeutic drugs used in these clinical trials include the platinum‐based chemotherapeutic drugs (e.g., cisplatin and http://www.guidetopharmacology.org/GRAC/LigandDisplayForward?ligandId=7433), http://www.guidetopharmacology.org/GRAC/LigandDisplayForward?ligandId=4793, and http://www.guidetopharmacology.org/GRAC/LigandDisplayForward?ligandId=6809. These drugs can cause severe side effects during therapy such as nephrotoxicity caused by cisplatin and a high prevalence of haematopoiesis suppression by gemcitabine (Manohar & Leung, 2018; Takei et al., 2017). Although some of the clinical trials were complete, none of the results was reported in detail (Table 1). The aim of this article is to highlight recent preclinical evidence on the potential of natural products as adjuvants in cancer therapy.

**Table 1:** Clinical trials for natural compounds or herbal medicines combining with chemotherapy

Recruitment status	Natural compounds	Drugs	Phase	Disease	Trial ID
Clinical trial for natural compound combinations					
Unknown	Curcumin	Gemcitabine http://www.guidetopharmacology.org/GRAC/LigandDisplayForward?ligandId=2892	III	Pancreatic cancer	NCT00486460
+	+	+	+	Colon cancer	NCT00295035
Clinical trial for herbal products combinations					
Completed	Teng‐Long‐Bu‐Zhong‐Tang	Oxaliplatin http://www.guidetopharmacology.org/GRAC/LigandDisplayForward?ligandId=6799	II	Colon cancer	NCT01975454
	TCM	http://www.guidetopharmacology.org/GRAC/LigandDisplayForward?ligandId=7154 Doxorubicin HCl	+	Breast cancer	NCT00028964
	+	Docetaxel http://www.guidetopharmacology.org/GRAC/LigandDisplayForward?ligandId=7105 Gemcitabine	+	NSCLC	NCT01780181
	+	Vinorelbine Platinum‐based chemotherapy	III	+	NCT01441752
	+	CDDP 5‐FU	II	Peritoneal carcinomatosis	NCT02638051
	Jin Fu Kang	Docetaxel	+	NSCLC	NCT00260026
Recruiting	TCM	Adjuvant chemotherapy	+	Breast cancer	NCT03797248
	+	Standard chemotherapy protocols	I	NSCLC	NCT02737735
Enrolling by invitation	Yiqi‐yangyin‐jiedu decoction	Gefitinib	III	Lung cancer	NCT02929693
Active, not recruiting	PHY906	http://www.guidetopharmacology.org/GRAC/LigandDisplayForward?ligandId=5711	I	Liver cancer	NCT01666756
Unknown	+	Gefitinib http://www.guidetopharmacology.org/GRAC/LigandDisplayForward?ligandId=4920 http://www.guidetopharmacology.org/GRAC/LigandDisplayForward?ligandId=7641	NA	Pulmonary adenocarcinoma	NCT01745302
	+	Lotrozole	NA	Polycystic ovary syndrome	NCT01431352

*Note*. For detail information about each clinical trial, see following website: https://clinicaltrials.gov/.

Abbreviations: +, The same with above cell; 5‐FU, 5‐Fluorouracil; CDDP, cisplatin; NA, not applicable; NSCLC, non‐small‐cell lung cancer; TCM, traditional Chinese medicine.

## 2 HERBAL COMPOUNDS WITH THE POTENTIAL TO SYNERGIZE WITH ANTITUMOR DRUGS

A synergistic effect is described as an increase in efficacy for a combination of components when compared with a single one (Pai, Cottrell, Kashuba, & Bertino, 2015). Data focusing on the toxic episodes of chemotherapy has led to the characterization of novel strategies, including the exploitation of natural compounds in combination therapies. The goals of including natural compounds in cancer chemotherapies are as follows: (a) to widen the therapeutic window of the chemotherapeutic drugs and (b) to decrease the occurrence of chemotherapy resistance (Ouyang et al., 2014). The next section will summarise herbal or folk medicines and natural compounds that act as chemosensitizers, chemoresistance reducers, or chemotherapeutic protectors, in clinical use.

### 2.1 Natural compounds acting as chemotherapeutic drug sensitizers

Chemosensitization refers to the potentiation of the tumoricidal effect of chemotherapeutic drugs by other low MW compounds, including making cancer cells more predisposed to chemotherapeutic drugs (Oliveira, Mendes, & Torchilin, 2017). The chemosensitizers can be natural products or synthetic compounds. This section will discuss the naturally sourced chemosensitizers that make cancer cells aware of responding therapeutic agents. http://www.guidetopharmacology.org/GRAC/LigandDisplayForward?ligandId=6785 is a natural alkaloid isolated from *Catharanthus roseus* that is currently used in acute lymphocytic lymphoma and neuroblastoma (Below & Das, 2019). However, due to its high cytotoxicity and narrow therapeutic window, it is restricted for further use, especially in paediatric malignancy (Parasramka, Talari, Rosenfeld, Guo, & Villano, 2017). Bahmani et al. (2018) found that another plant extract from *Centaurea albonitens*, could significantly enhance the cytotoxicity of vincristine against leukaemia cell lines without increasing toxicity to normal cells. To reduce the cardiotoxicity and resistance due to doxorubicin, numerous plant extracts have been used with doxorubicin, in screening synergistic effects. So far, an aqueous extract of *Solanum nigrum* Linn. was shown to potentiate doxorubicin against colorectal cancer and ovarian cancer through autophagy induction (Tai et al., 2013; C. W. Wang et al., 2015). Based on the same idea, polysaccharides isolated from *Agrocybe aegerita* and aqueous extract of *S. nigrum* Linn. were found to increase cytotoxicity of http://www.guidetopharmacology.org/GRAC/LigandDisplayForward?ligandId=4789 (5‐FU) against oesophageal carcinoma, ovarian cancer, and colorectal cancer via regulating pro‐inflammatory cytokine such as http://www.guidetopharmacology.org/GRAC/LigandDisplayForward?ligandId=5074 and IFN‐γ (Ji, Zheng, Ye, Wu, & Chen, 2013; Tai et al., 2013; C. W. Wang et al., 2015). For increasing the anti‐cancer effects of paclitaxel, three natural phenolic acids (http://www.guidetopharmacology.org/GRAC/LigandDisplayForward?ligandId=5155, rosmarinic acid, and ursolic acid) were used with paclitaxel in ex vivo breast cancer cells, and found to promote cytotoxicity these cells by modulating the tumour micro‐environment (Carranza‐Torres et al., 2015). Such results show that it is possible to increase the cytotoxic effects of known anti‐cancer agents, with natural compounds. There are numerous studies focusing on synergism between herbal compounds and cancer therapeutic drugs, both in vitro and in vivo. A large percentage of the natural compounds are flavonoids and phenolics, which implies that flavonoids and phenolics have more potential than other subgroups. However, curcumin is the most studied natural compound (Table 2). These studies cover the most prevalent and fatal cancers, for example, lung cancer, breast cancer, and colorectal cancer. Interestingly, these studies focused on curcumin potentiating chemotherapeutic efficacy (by http://www.guidetopharmacology.org/GRAC/LigandDisplayForward?ligandId=7624, 5‐FU, doxorubicin, and radiation) have shown that curcumin promotes chemotherapy through regulating the expression or activity of the transcription factor NF‐κB. This finding implies that curcumin might target upstream signalling modulators of NF‐κB or NF‐κB itself (Table 2). Most of these herbal enhancers for promoting cytotoxicity of chemotherapeutic agents exert their functions via targeting the stress–stimuli response, that is to oxidative stress and particularly NF‐κB, which seems to be an indicator for determining the potency of chemotherapeutic cytotoxicity. When taken together, natural compounds or herbal products have a high potential to support chemotherapeutic drugs to fight cancer cells.

**Table 2:** Herbal compounds act as an enhancer of cancer therapy

Structure subclass	Natural compound	Chemotherapeutic drug	Cancer	Signal pathway	Reference^a^
Cell death via specific signalling pathway					
Alkaloid	3,3′‐Diindolylmethane	Cisplatin	Ovary	http://www.guidetopharmacology.org/GRAC/ObjectDisplayForward?objectId=2994/Akt	(Zou, Xu, Li, Zhang, & Fan, 2018)
	Berberine	Radiation	Esophagus	Rad51	(Liu et al., 2011)
	Ethoxysanguinarine	Cisplatin	Lung	CIP2A	(Liu, Ma, Wen, Cheng, & Zhou, 2014)
	http://www.guidetopharmacology.org/GRAC/LigandDisplayForward?ligandId=224	+	Liver	NF‐κB/AP‐2β	(Hao et al., 2017)
	Neferine	Doxorubicin	Lung	http://www.guidetopharmacology.org/GRAC/ObjectDisplayForward?objectId=1875/ROS	(Poornima, Kumar, Weng, & Padma, 2014)
	http://www.guidetopharmacology.org/GRAC/LigandDisplayForward?ligandId=10212	Cisplatin	Ovary	HIF‐1α	(Su et al., 2011)
	Piperlongumine	Doxorubicin	Prostate	http://www.guidetopharmacology.org/GRAC/ObjectDisplayForward?objectId=1383	(Piska et al., 2019)
Capsaicinoid	http://www.guidetopharmacology.org/GRAC/LigandDisplayForward?ligandId=2486	Radiation	Prostate	NF‐κB	(Venier et al., 2015)
Diarylheptanoid	Curcumin	5‐FU	Gastric	NF‐κB	(Kang et al., 2016)
	+	Carboplatin	Lung	Akt/NF‐κB	(Kang et al., 2015)
	+	+	Breast	FEN1	(Zou et al., 2018)
	+	+	Colorectal	endoG/NF‐κB	(Wang, Liu, & Su, 2014)
	+	+	Lymphoma	Rad51, apoptosis‐Caspase	(Zhao et al., 2018)
	+	+	Neuroblastoma	Uniquitin	(D'Aguanno et al., 2012)
	+	+	Ovary	c‐Myb/STAT3/NF‐κB	(Tian, Tian, Qiao, Li, & Zhang, 2019)
	+	Doxorubicin	Gastric	NF‐κB	(Yu, Wu, Dai, Yu, & Si, 2011)
	+	Radiation	Prostate	miR‐143	(Liu, Li, Wang, & Luo, 2017)
	+	Rhtrail	Breast	http://www.guidetopharmacology.org/GRAC/ObjectDisplayForward?objectId=1880/http://www.guidetopharmacology.org/GRAC/FamilyDisplayForward?familyId=889	(Park, Cho, Andera, Suh, & Kim, 2013)
Diterpenoid	Adenanthin	http://www.guidetopharmacology.org/GRAC/LigandDisplayForward?ligandId=2779	Leukaemia	Prx‐1/C/EBP	(Wei et al., 2016)
	Cryptotanshinone	Cisplatin	Ovary	http://www.guidetopharmacology.org/GRAC/LigandDisplayForward?ligandId=4470 and http://www.guidetopharmacology.org/GRAC/ObjectDisplayForward?objectId=1633	(Jiang et al., 2017)
	+	http://www.guidetopharmacology.org/GRAC/LigandDisplayForward?ligandId=2770	Oral	http://www.guidetopharmacology.org/GRAC/FamilyIntroductionForward?familyId=581/STAT3 e‐Cadherin/p53/β‐catenin	(Wang et al., 2017)
Flavonoid	(−)‐Epicatechin	Radiation	Pancreas/Glioma	http://www.guidetopharmacology.org/GRAC/ObjectDisplayForward?objectId=1987/p21	(Elbaz, Lee, Antwih, Liu, Huttemann, & Zielske, 2014)
	Formononetin	Doxorubicin	Gastric	http://www.guidetopharmacology.org/GRAC/ObjectDisplayForward?objectId=2660	(Liu et al., 2015)
	Icariin	5‐FU	Colorectal	NF‐κB	(Shi et al., 2014)
	http://www.guidetopharmacology.org/GRAC/LigandDisplayForward?ligandId=5215	Cisplatin	Bile duct	http://www.guidetopharmacology.org/GRAC/FamilyDisplayForward?familyId=781_3_K/Akt/http://www.guidetopharmacology.org/GRAC/ObjectDisplayForward?objectId=2109/SREP	(Lim, Yang, Bazer, & Song, 2016)
	http://www.guidetopharmacology.org/GRAC/LigandDisplayForward?ligandId=10298	Paclitaxel	Prostate	PI_3_K/Akt and http://www.guidetopharmacology.org/GRAC/FamilyDisplayForward?familyId=514	(Lim, Park, Bazer, & Song, 2017)
	WYC02	Cisplatin	Multi cancer	ATM	(Wang et al., 2012)
	http://www.guidetopharmacology.org/GRAC/LigandDisplayForward?ligandId=5346	Rhtrail	Breast	http://www.guidetopharmacology.org/GRAC/LigandDisplayForward?ligandId=5188/DR5	(Manouchehri, Turner, & Kalafatis, 2018)
	Silibinin	5‐FU	Colorectal	PI_3_K/MAPK/http://www.guidetopharmacology.org/GRAC/LigandDisplayForward?ligandId=5371/nanog/CD44v6	(Patel et al., 2018)
Isoprenoid	http://www.guidetopharmacology.org/GRAC/LigandDisplayForward?ligandId=2771	+	Liver	NF‐κB	(Zhang et al., 2011)
Macrolide	Elaiophylin	Cisplatin	Ovary	http://www.guidetopharmacology.org/GRAC/ObjectDisplayForward?objectId=2348	(Zhao et al., 2015)
Monoterpenoid	http://www.guidetopharmacology.org/GRAC/LigandDisplayForward?ligandId=6413	+	Oesophagus	PI_3_K/Akt	(Meng et al., 2018)
Organosulfur	http://www.guidetopharmacology.org/GRAC/LigandDisplayForward?ligandId=6569	Doxorubicin	Ovary	SFN, http://www.guidetopharmacology.org/GRAC/ObjectDisplayForward?objectId=3055	(Pastorek et al., 2015)
Phenolic	http://www.guidetopharmacology.org/GRAC/LigandDisplayForward?ligandId=6999	Radiation	Prostate	γ‐H2AX	(Yao et al., 2015)
	Caffeic acid	http://www.guidetopharmacology.org/GRAC/LigandDisplayForward?ligandId=4779	Cervix	http://www.guidetopharmacology.org/GRAC/ObjectDisplayForward?objectId=1540/TCA cycle	(Tyszka‐Czochara, Konieczny, & Majka, 2017)
	+	+	+	SNAI1/MMP‐9	(Tyszka‐Czochara, Lasota, & Majka, 2018)
	Capsaicin	Docetaxel	Prostate	http://www.guidetopharmacology.org/GRAC/ObjectDisplayForward?objectId=2497/PI_3_K/Akt/mTOR http://www.guidetopharmacology.org/GRAC/ObjectDisplayForward?objectId=2212/AMPK	(Sanchez, Bort, Mateos‐Gomez, Rodriguez‐Henche, & Diaz‐Laviada, 2019)
	Dicoumarol	Doxorubicin	Urinary tract	NADPH quinone oxidoreductase	(Matsui et al., 2010)
	Emodin	http://www.guidetopharmacology.org/GRAC/LigandDisplayForward?ligandId=1016	Breast	Ras/ERK PI_3_K/mTOR	(Tseng et al., 2017)
Polyyne	Falcarindiol	5‐FU	Colorectal	ER stress	(Jin et al., 2012)
Susquiterpenoid	Heteronemin	Cytarabine	Leukaemia	http://www.guidetopharmacology.org/GRAC/FamilyDisplayForward?familyId=897 farnesylation	(Saikia et al., 2018)
	β‐Eudesmol	Doxorubicin 5‐FU	Bile duct	NADPH quinone oxidoreductase	(Srijiwangsa, Ponnikorn, & Na‐Bangchang, 2018)
Phytosteroid	Polyphyllin D	Cisplatin	Ovary	18 unique genes	(Al Sawah et al., 2015)
	Tenacigenin B derivative	Paclitaxel	Ovary	Inhibit Cytochrome P450	(Xie et al., 2019)
Stilbenoid	Resveratrol	Cisplatin	Lung	Mitochondrial depolarization	(Ma et al., 2015)
	+	Doxorubicin	Breast	HSP‐27	(Diaz‐Chavez et al., 2013)
	+	+	+	Carbonyl reductase 1	(Ito et al., 2013)
Tetrahydrofuran	Acetogenin	Doxorubicin	Ovary	Mitochondrial complex I	(Tormo et al., 2003)
Tripyrrole	Prodigiosin	Paclitaxel	+	http://www.guidetopharmacology.org/GRAC/ObjectDisplayForward?objectId=2795	(Ho et al., 2009)
	+	Doxorubicin	Oral	Doxorubicin accumulation	(Lin & Weng, 2018)
Triterpenoid	Brusatol	5‐FU	Pancreas	e‐cadherin/Twist/vimentin/NF‐κB	(Lu, Lai, Leung, Leung, Li, & Lin, 2017)
Triterpenoid	http://www.guidetopharmacology.org/GRAC/LigandDisplayForward?ligandId=10386	Cisplatin	Lung	FANCD2	(Wang, Liu, Cheng, & Zhou, 2015)
	+	Tanespimycin	Glioblastoma	http://www.guidetopharmacology.org/GRAC/ObjectDisplayForward?objectId=618, http://www.guidetopharmacology.org/GRAC/ObjectDisplayForward?objectId=2540, Hsp90	(Boridy, Le, Petrecca, & Maysinger, 2014)
Xanthonoid	Formononetin	Metformin	Breast	ERK1/2/http://www.guidetopharmacology.org/GRAC/ObjectDisplayForward?objectId=2844	(Xin, Wang, Ren, & Guo, 2019)
Via apoptosis or autophagy					
Alkaloid	Berberine	Sorafenib	Liver	Apoptosis‐Intrinsic	(Huang et al., 2018)
	Indole‐3‐carbinol	Cisplatin	Ovary	+	(Taylor‐Harding et al., 2012)
	+	Doxorubicin	Cervix	+	(Adwas, Elkhoely, Kabel, Abdel‐Rahman, & Eissa, 2016)
Carotenoid	Bixin	+	Acute leukaemia	Apoptosis	(Santos, Almeida, Antunes, & Bianchi, 2016)
Diarylheptanoid	Curcumin	Cisplatin	Lung	Apoptosis‐Intrinsic	(Baharuddin et al., 2016)
	+	+	Oral	Apoptosis‐Intrinsic	(Chen et al., 2018)
	+	Sorafenib	Liver	Apoptosis‐Intrinsic	(Bahman, Abaza, Khoushiash, & Al‐Attiyah, 2018)
Diterpenoid	Crassin	Doxorubicin	Breast	Apoptosis‐ROS	(Richards, Vellanki, Smith, & Hopkins, 2018)
	Ent‐kaurane‐type diterpenoids	+	Liver	Apoptosis	(Pham, Iscache, Pham, & Gairin, 2016)
Flavonoid	Eupatorin	+	Colorectal	Apoptosis‐Intrinsic	(Namazi Sarvestani, Sepehri, Delphi, & Moridi Farimani, 2018)
	http://www.guidetopharmacology.org/GRAC/LigandDisplayForward?ligandId=4285	Cisplatin	Lung	Apoptotic/MMPs	(Ma, Wang, Nan, Li, Wang, & Jin, 2016)
	Salvigenin	Doxorubicin	Colorectal	Apoptosis‐Intrinsic	(Namazi Sarvestani, Sepehri, Delphi, & Moridi Farimani, 2018)
Lignan	Enterolactone	+	Breast	Apoptosis	(Di, De Silva, Krol, & Alcorn, 2018)
	Secoisolariciresinol	+	+	+	(Di, De Silva, Krol, & Alcorn, 2018)
Organosulfur	http://www.guidetopharmacology.org/GRAC/LigandDisplayForward?ligandId=4822	Temozolomide	Colorectal	Autophagy	(Goder et al., 2015)
	Alyssin	5‐FU	Colorectal	Apoptosis‐Extrinsic	(Milczarek et al., 2018)
Phenolic	http://www.guidetopharmacology.org/GRAC/LigandDisplayForward?ligandId=7062	Photodynamic therapy	Ehrlich	Apoptosis‐Intrinsic	(Joy, Nishanth Kumar, Soumya, Radhika, Vibin, & Abraham, 2014)
Phenolic	Nordihydroguaiaretic acid	Cisplatin	Breast	ROS	(Mundhe, Kumar, Ahmed, Jamdade, Mundhe, & Lahkar, 2015)
Phenolic	http://www.guidetopharmacology.org/GRAC/LigandDisplayForward?ligandId=10302	+	Lung	Apoptosis‐Intrinsic	(Xu et al., 2013)
Sesquiterpenes	Trans‐nerolidol	Doxorubicin	Breast	doxorubicin accumulation	(Hanusova et al., 2017)
	β‐Caryophyllene oxide	+	+	+	(Hanusova et al., 2017)
	β‐Elemene	Cisplatin	Lung/Brain/Breast/ Cervix/Ovary/Colorectal	Apoptpsis‐Intrinsic & Extrinsic	(Li et al., 2013)
Stilbenoid	Resveratrol	Sorafenib	Liver	Apoptosis‐Intrinsic	(Bahman, Abaza, Khoushiash, & Al‐Attiyah, 2018)
Triterpenoid	Withaferin A	Radiation	Lymphoma	Apoptosis‐ROS, Bcl‐2	(Yang, Choi, Kim, Choi, & Kwon, 2011)
	Acetyl‐11‐keto‐β‐boswellic acid	+	Glioblastoma	Apoptosis‐Intrinsic	(Conti et al., 2018)
Xanthonoid	Forbesione	5‐FU	Bile duct	Apoptosis‐Intrinsic	(Boueroy et al., 2017)
	Gambogic acid	Doxorubicin	Ovary	Apoptosis‐ROS	(Wang & Yuan, 2013)
	Kaempferol	Sorafenib	Liver	Apoptosis‐Intrinsic	(Bahman, Abaza, Khoushiash, & Al‐Attiyah, 2018)
Reducing chemoresistance via specific mechanism					
Alkaloid	Aaptamine	Cisplatin	Embryonal carcinoma	http://www.guidetopharmacology.org/GRAC/ObjectDisplayForward?objectId=598, p53, eIF5A hypusination	(Dyshlovoy et al., 2014)
	Demethyloxyaaptamine	+	+	TNF	(Dyshlovoy et al., 2014)
	Isoaaptamine	+	+	myc, p53, TNF	(Dyshlovoy et al., 2014)
	Sinapine	Doxorubicin	Colorectal	http://www.guidetopharmacology.org/GRAC/ObjectDisplayForward?objectId=1811‐FRS2α‐ERK1/2	(Guo, An, Feng, Liu, Wang, & Zhang, 2014)
Diarylheptanoid	Curcumin	Cisplatin	Lung	http://www.guidetopharmacology.org/GRAC/ObjectDisplayForward?objectId=2029/BRCA	(Chen, Li, Jiang, Lan, & Chen, 2015)
	+	+	Ovary	MEG3, miR‐214	(Zhang, Liu, Xu, & Li, 2017)
	+	+	+	miR‐186	(Tang, Zhang, & Du, 2010)
Flavonoid	Isoliquiritigenin	+	Oral	ALDH1, http://www.guidetopharmacology.org/GRAC/LigandDisplayForward?ligandId=9981, GRP78	(Hu, Yu, Hsieh, Liao, Lu, & Chu, 2017)
	http://www.guidetopharmacology.org/GRAC/LigandDisplayForward?ligandId=9738	Paclitaxel	Ovary	Akt/http://www.guidetopharmacology.org/GRAC/ObjectDisplayForward?objectId=3008/http://www.guidetopharmacology.org/GRAC/FamilyDisplayForward?familyId=578	(Yang et al., 2012)
	Wogonin	Doxorubicin	Breast	Nrf2	(Zhong et al., 2013)
Lignan	Silybin	+	Colon	GLUT1	(Catanzaro et al., 2018)
Nucleoside	Clitocine	+	Liver	NF‐κB	(Sun et al., 2012)
Organosulfur	Sulforaphane	Cisplatin	Ovary	HIF‐1α	(Pastorek et al., 2015)
Phenol	Phenylethyl isothiocyanate	+	In vivo	http://www.guidetopharmacology.org/GRAC/ObjectDisplayForward?objectId=2847 glutathionylation	(Li et al., 2016)
	Emodin	Doxorubicin	Lung	Anthracycline reductases	(Hintzpeter, Seliger, Hofman, Martin, Wsol, & Maser, 2016)
Steroid	Cucurbitacin b	+	Gastric	CIP2A/PP2A/mTORC1	(Liu et al., 2017)
Triterpenoid	Adcx	Paclitaxel	Liver	Akt/autophagy	(Sun et al., 2017)
	Polyphyllin I	Erlotinib	Lung	http://www.guidetopharmacology.org/GRAC/LigandDisplayForward?ligandId=4998/STAT3	(Lou, Chen, Zhu, Deng, Wu, & Wang, 2017)
Via inhibiting drug efflux					
Alkaloid	Cinchonine	Paclitaxel	Uterine		(Lee et al., 2011)
	Hydrocinchonine	Paclitaxel	Uterine		(Lee et al., 2011)
	http://www.guidetopharmacology.org/GRAC/LigandDisplayForward?ligandId=2342	+	+		(Lee et al., 2011)
Diarylheptanoid	Curcuminoid	Doxorubicin	Leukaemia		(Xu, Tian, & Shen, 2013)
Diterpenoid	Tanshinone IIA	Doxorubicin	Gastric		(Xu et al., 2018)
Flavonoid	http://www.guidetopharmacology.org/GRAC/LigandDisplayForward?ligandId=2829	Daunorubicin	Breast		(Zhang, Sagawa, Arnold, Tseng, Wang, & Morris, 2010)
	Glabridin	Doxorubicin	+		(Qian et al., 2019)
Lignan	Matairesinol	Doxorubicin	Colon		(Su, Cheng, & Wink, 2015)
	+	+	Leukaemia		(Su, Cheng, & Wink, 2015)
Monoterpene	Borneol‐peg‐np	Paclitaxel	Ovary		(Zou et al., 2017)
Triterpenoid	Maslinic acid	+	Diarthrosis/smooth muscle		(Villar et al., 2014)
	http://www.guidetopharmacology.org/GRAC/LigandDisplayForward?ligandId=3306	+	+		(Villar et al., 2014)
	Ursolic acid	Doxorubicin	Breast		(Zong, Cheng, Liu, Pi, Liu, & Song, 2019)
Xanthone	Forbesione	Doxorubicin	Bile duct	NF‐κB & p‐Glycoprotein	(Hahnvajanawong et al., 2014)
	Isomorellin	+	+		(Hahnvajanawong et al., 2014)
Xanthonoid	Mangiferin	+	Breast	p‐Glycoprotein, MRP‐1, http://www.guidetopharmacology.org/GRAC/ObjectDisplayForward?objectId=792	(Louisa, Soediro, & Suyatna, 2014)
	Gambogic acid	Multidrugs	Multi‐cancer	p‐Glycoprotein	(Wang et al., 2013)

*Note*. Intrinsic: Bcl‐2/Bcl‐XL/caspase‐3, 9; Extrinsic: DR/Bid/caspase‐3, 7, 8; +, the same with above cell.

a For Reference list, see Data S1.

### 2.2 Herbal compounds reduce resistance against cancer therapy

Clinically, herbal compounds can reduce resistance against cancer therapies, and this has become a critical concern. Up to now, drug resistance (excluding radiation‐resistance) in cancer cells remains the most challenging aspect of cancer treatment, especially in NSCLC and prostate cancer (Chang, 2011; Wade & Kyprianou, 2018). Such resistance in cancers reveals a transformation of cancer cells from drug susceptible to resistant, which leads to higher toxicity and expenditures in treatments (Housman et al., 2014; Zheng, 2017). About 90% of treatment failures in recurrent cancer therapy and 80–90% of cancer death is strongly correlated to cancer resistance (Mansoori, Mohammadi, Davudian, Shirjang, & Baradaran, 2017; Yuan et al., 2017).

Prevailing mechanisms of chemoresistance are classified into seven phases: drug flux, DNA damage repair, cell death inhibition, epithelial‐mesenchymal transition (EMT), drug target alteration, drug inactivation, and epigenetics (Housman et al., 2014), and notably, drug flux is the most concerned issue in this topic. Cancer cells pump chemotherapeutic agents out of the cells using the http://www.guidetopharmacology.org/GRAC/FamilyDisplayForward?familyId=153 (MRPs, also known as MDR or the ABCC family of transporters) and the Hedgehog receptor Patched 1 (protein patched homolog 1, PTCH1), which reduces drug accumulation within cancer cells and, thereby, lower drug efficacy (Amiri‐Kordestani, Basseville, Kurdziel, Fojo, & Bates, 2012; Bidet et al., 2012). MRPs, particularly http://www.guidetopharmacology.org/GRAC/ObjectDisplayForward?objectId=779 (also known as p‐glycoprotein, P‐gp), are found over‐expression in recurrent cancer cells and which over‐expression is associated with poor prognosis (Chen et al., 2016; W. Li et al., 2016). PTCH1 is a newly discovered drug efflux transporter also found to be overexpressed in many metastatic cancers (Hasanovic & Mus‐Veteau, 2018). In addition to drug efflux, PTCH1 also acts as a receptor in Hedgehog/Gli signalling pathway that activates http://www.guidetopharmacology.org/GRAC/ObjectDisplayForward?objectId=239 (Smo)/Gli transduction and leads to growth factor expression (Armas‐Lopez, Zuniga, Arrieta, & Avila‐Moreno, 2017; Rimkus, Carpenter, Qasem, Chan, & Lo, 2016). Some chemotherapeutic agents specially target growth factor signalling, for example, http://www.guidetopharmacology.org/GRAC/LigandDisplayForward?ligandId=4941 (Iressa®, http://www.guidetopharmacology.org/GRAC/ObjectDisplayForward?objectId=1797 inhibitor), http://www.guidetopharmacology.org/GRAC/LigandDisplayForward?ligandId=5082 (Herceptin®, http://www.guidetopharmacology.org/GRAC/ObjectDisplayForward?objectId=2019/neu inhibitor), and http://www.guidetopharmacology.org/GRAC/LigandDisplayForward?ligandId=6771 (Avastin®, http://www.guidetopharmacology.org/GRAC/FamilyDisplayForward?familyId=324 inhibitor; National Cancer Institute, 2002a, 2002b). However, cancer cells turn on EMT, which leads to anoikis resistance and continuous activation of growth factor signalling during cancer invasion (J. Wang et al., 2016). EMT‐induced chemoresistance has been identified in several cancer types, including lung cancer, prostate cancer, and breast cancer (Fischer et al., 2015; J. Huang, Li, & Ren, 2015; Wade & Kyprianou, 2018).

**Figure 1:**
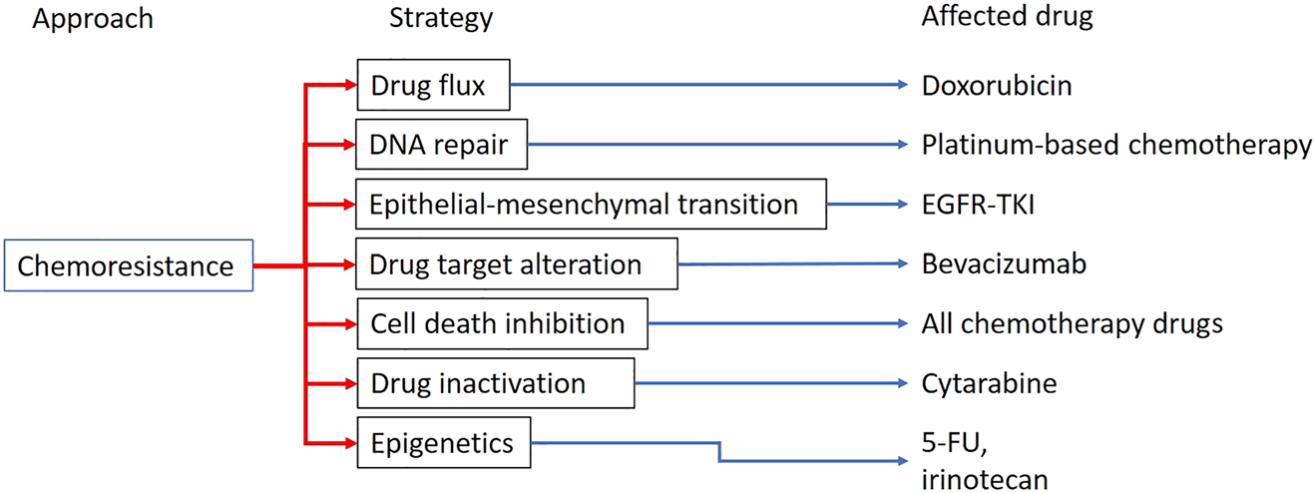
Recent‐known mechanism of chemoresistance

Numerous cancer drugs, including platinum‐based chemotherapeutic drugs, http://www.guidetopharmacology.org/GRAC/LigandDisplayForward?ligandId=7242, http://www.guidetopharmacology.org/GRAC/LigandDisplayForward?ligandId=6823, and doxorubicin, belong to the group known as DNA damage agents (Cheung‐Ong, Giaever, & Nislow, 2013). Therefore, DNA repairing capacity would directly affect these cancer drugs' effects (Nagel et al., 2017; Sakthivel & Hariharan, 2017). However, Wang et al. have explored the involvement of Wip1, which is an inhibitor of the http://www.guidetopharmacology.org/GRAC/ObjectDisplayForward?objectId=1934 kinase‐mediated DNA repairing system, in cancer resistance of oral squamous cell carcinoma (OSCC). Wip1 activation is thought to potentiate the cytotoxicity of cisplatin against OSCC (L. Wang, Mosel, Oakley, & Peng, 2012). Unexpectedly, a positive correlation between Wip1 expression and cisplatin resistance in OSCC has now emerged (L. Wang et al., 2012). Thus, both positive and negative relationships between DNA repairing mechanisms and chemoresistance have been found and further investigation is needed to clarify the characteristics of DNA repairing mechanisms in chemoresistance. Figure 1 summarizes the above discussion, showing seven mechanisms of chemoresistance proposed, and giving examples of chemotherapeutic drugs affected by particular chemoresistance mechanisms.

Recent studies on the effects of natural compounds against chemoresistance show that they inhibit MDR protein activity or further reduce MDR protein expressions (Turrini, Ferruzzi, & Fimognari, 2014). Table 2 lists these MDR inhibitors and compounds acting through other mechanisms. This Table shows that MDR inhibition accounts for the highest rate. Interestingly, silybin, a natural lignan isolated from *Silybum marianum*, allows doxorubicin to overcome drug resistance in colorectal cancer by inhibiting http://www.guidetopharmacology.org/GRAC/ObjectDisplayForward?objectId=875 (GLUT1) expression. GLUT1 expression could be regulated by the Wnt/http://www.guidetopharmacology.org/GRAC/LigandDisplayForward?ligandId=5371 signalling pathway that has been identified as a cisplatin resistance promoter through the ATM‐mediated signalling pathway in laryngeal squamous cell carcinoma cells (L. Wang et al., 2019). Eleven different polyoxypregnanes isolated from *Marsdenia tenacissima* can combat doxorubicin resistance in multidrug resistance cancer cell lines via inhibition of ABC transporters (To et al., 2017). A series of bisbenzylisoquinoline alkaloids inhibit the transporter P‐gp, which leads to high doxorubicin accumulation in MCF‐7/ADR breast cancer cell to provide much increased cytotoxicity (Sun & Wink, 2014). Investigation of the antitumor activity of six ergot alkaloids from *Claviceps purpurea*, showed that these ergot alkaloids might bypass chemoresistance mechanisms through unknown signalling pathways in multiple cancers (Mrusek, Seo, Greten, Simon, & Efferth, 2015). Additionally, ellagic acid and http://www.guidetopharmacology.org/GRAC/LigandDisplayForward?ligandId=8741 prevented induction of resistance in ovarian cancer towards cisplatin (Engelke, Hamacher, Proksch, & Kassack, 2016). (Z)‐3,4,3′,5′‐tetramethoxystilbene, a stilbenoid, increased antitumor efficacy of cisplatin in cisplatin‐resistant osteosarcoma cells in in vitro and in vivo (H. Xu, 2016), and another stilbenoid, resveratrol, increased cisplatin uptake and efficacy (Osman et al., 2015). β‐Phenylethyl isothiocyanate and http://www.guidetopharmacology.org/GRAC/LigandDisplayForward?ligandId=2428 down‐regulated intracellular http://www.guidetopharmacology.org/GRAC/LigandDisplayForward?ligandId=6737 level and concurrently reversed resistance to doxorubicin and cisplatin in resistant‐uterine sarcoma cells (Angelini, Conti, Ciofani, Cuccurullo, & Di Ilio, 2013; W. J. Wu et al., 2013). Taken together, the data suggest herbal compounds exert beneficial effects in the treatment of recurrent cancers, when combined with current therapies.

### 2.3 Chemopreventive effect of herbal compounds

The non‐selective character of most chemotherapeutic drugs usually initiates systemic symptoms as adverse or side effects during therapy (de Oliveira Junior et al., 2018). These adverse effects include cardiotoxicity, nephrotoxicity, hepatotoxicity, and peripheral neuropathy (Duwe et al., 2017; Ma, Kavelaars, Dougherty, & Heijnen, 2018; Santoni et al., 2017; Sharbaf et al., 2017). Sometimes, the adverse effects severely affect the daily quality of life for patients (X. Wu et al., 2016). Many of the adverse effect of chemotherapeutic drugs are caused by the drug itself and its metabolites, usually by inducing ROS formation (Varricchi et al., 2018). Accordingly, a study of doxorubicin metabolism indicated that it was the main cause of doxorubicin‐induced cardiomyopathy, through the generation of toxic intermediates and ROS, leadong to the apoptosis of cardiomyocytes (Renu, Abilash, Tirupathi Pichiah, & Arunachalam, 2018). In addition, many naturally sourced antioxidants are present in plant and herbal sources (D. P. Xu et al., 2017). Therefore, herbal compounds intended to alleviate the adverse effects of chemotherapeutic drugs have been assessed for antioxidation or ROS scavenger effects, in vitro and in vivo. One study in vitro, using cardiomyocytes and doxorubicin indicated that saffron extract could activate http://www.guidetopharmacology.org/GRAC/FamilyDisplayForward?familyId=285/http://www.guidetopharmacology.org/GRAC/ObjectDisplayForward?objectId=1525 and http://www.guidetopharmacology.org/GRAC/FamilyDisplayForward?familyId=514 pathways resulting in decreased cardiomyocytic apoptosis (Chahine, Nader, Duca, Martiny, & Chahine, 2016). In another report focused on irinotecan toxicity, the TCM, Gegen Qinlian decoction, ameliorated gut inflammation by activating the http://www.guidetopharmacology.org/GRAC/ObjectDisplayForward?objectId=2757/http://www.guidetopharmacology.org/GRAC/ObjectDisplayForward?objectId=3057 pathway and might result in up‐regulation of tight junction and down‐regulation of inflammatory cytokines (Y. Wu et al., 2019). Other studies have also shown that anthocyanin from black rice tested in vitro with on cardiomyocytes attenuated cardiotoxicity via http://www.guidetopharmacology.org/GRAC/ObjectDisplayForward?objectId=620/http://www.guidetopharmacology.org/GRAC/ObjectDisplayForward?objectId=2021 and HSF‐1 signalling pathways, and http://www.guidetopharmacology.org/GRAC/LigandDisplayForward?ligandId=7002 (EGCG) reduceds NADPH‐cytochrome P‐450 reductase activity (the key enzyme of doxorubicin toxicity; Dudka et al., 2005; P. C. Huang et al., 2016). In a cell model, http://www.guidetopharmacology.org/GRAC/LigandDisplayForward?ligandId=10047 and 3,3′‐di‐indolylmethane were cardioprotective in mouse models through the Nrf2/ARE pathway as well (Adwas et al., 2016; Hajra, Basu, Singha Roy, Patra, & Bhattacharya, 2017).

Several herbal compounds including nordihydroguaiaretic acid, eriodictyol‐7‐O‐glucoside, and http://www.guidetopharmacology.org/GRAC/LigandDisplayForward?ligandId=2499 ameliorated cisplatin‐induced renal injury, in vitro and in vivo (Hosseinimehr et al., 2015; Hu, Zhang, Wang, Lou, & Ren, 2012; Mundhe et al., 2015). Resveratrol, http://www.guidetopharmacology.org/GRAC/LigandDisplayForward?ligandId=2826, and epigallocatechin‐3‐gallate attenuated apoptosis of haematopoietic cells via reducing DNA damage (Alotaibi, Bhatnagar, Najafzadeh, Gupta, & Anderson, 2013; Olas & Wachowicz, 2004; Sonaa, Usha, & Ja In, 2013). Again, in photodynamic therapy research, *Pinus halepensis* bark extract prevented the photosensitivity in SCID mice model (Petri et al., 2012). Coniferyl aldehyde, found in wine, reduced radiation damage via phosphorylation of HSF‐1 and further increased the activation of ERK1/2 (S. Y. Kim, Lee, Nam, Seo, & Lee, 2015). All these experiments assessed the benefits of the chemoprotective ability of herbal compounds in cancer therapy, derived from a reduction of side effects and a consequent reduction in dose. Nonetheless, drug–herbal interactions, leading to injury may also be important dis‐advantages of combination therapy.

## 3 HERBAL TOXICITIES AND FUTURE REMARKS

An increasing number of cases require closer attention to the additional toxicity induced by the herbal component or by the herbal–drug combination (Table 3). In these examples of herbal‐induced drug injury, some popular formulations or compounds are included, such as curcumin and chokeberry, which is usually used for increasing patient stamina to overcome the adverse effects of cancer therapy. A meta‐analysis has collected 97 herbal‐induced toxicity cases in Korea and found that both single and multiple herbal preparations could induce hepatocellular toxicity, including *Polygonum multiflorum* and *Dictamnus dasycarpus* (W. J. Lee, Kim, Lee, & Son, 2015). A following review collects studies about monoterpene‐ and susquiterpenes‐induced hepatotoxicity and summarize that some common terpenes (e.g. http://www.guidetopharmacology.org/GRAC/LigandDisplayForward?ligandId=2422 and limonene) might injure liver through generating ROS and imparing antioxidant defenses (Zarybnicky, Bousova, Ambroz, & Skalova, 2018). In addition to ROS generation, another hepatotoxic mechanism is simultaneously observed through the modulation of cytochrome P450 (Brewer & Chen, 2017). Coumarins, furanocoumarins, (−)‐epigallocatechin‐3‐gallate, and http://www.guidetopharmacology.org/GRAC/LigandDisplayForward?ligandId=2489 have all demonstrated a potent inhibition of cytochrome P450 isoforms, especially http://www.guidetopharmacology.org/GRAC/ObjectDisplayForward?objectId=1337 which is a key enzyme for oral drug detoxification, and MDRs, which may be inhibited and thus prolong the *t*_1/2_ of the drug (Brewer & Chen, 2017; Pal & Mitra, 2006; Shamsi, Tran, Tan, Tan, & Lim, 2017). These findings of toxicity from using herbal medicine for cancer therapy suggest that precautions should be taken against the herb‐induced or drug‐induced liver injury.

**Table 3:** Natural compounds as potential adjuvants to cancer therapy: Unpredictable adverse events

Herbal compounds	Chemotherapeutic drugs	Cancer or normal cell type	Adverse effect and relevant mechanism	Reference^a^
Curcumin	Doxorubicin	Cardiac muscle cells	Apoptosis‐ROS	(Hosseinzadeh, Behravan, Mosaffa, Bahrami, Bahrami, & Karimi, 2011)
	http://www.guidetopharmacology.org/GRAC/LigandDisplayForward?ligandId=6815	Cervix/Breast/Colorectal	Offset cancer cell death via http://www.guidetopharmacology.org/GRAC/FamilyDisplayForward?familyId=331/γ‐H2AX	(Saleh, El‐awady, Eissa, & Abdel‐Rahman, 2012)
http://www.guidetopharmacology.org/GRAC/LigandDisplayForward?ligandId=4520	Taxane	Breast cancer	Increase peripheral neuropathy	(Hershman et al., 2013)
Chokeberry	http://www.guidetopharmacology.org/GRAC/LigandDisplayForward?ligandId=2774	Liposarcoma	Induce rhabdomyolysis	(Strippoli, Lorusso, Albano, & Guida, 2013)
Bu Zhong Yi Qi Wan	http://www.guidetopharmacology.org/GRAC/LigandDisplayForward?ligandId=7301/radiation	Glioblastoma	Induce acute liver toxicity	(Melchardt et al., 2014)

a For Reference list, see Data S2.

Accordingly, the best fit between possible adverse effect and anticancer efficacy is urgently needed in terms of clinical application. The balance between adverse effect and anticancer efficacy can be discussed at two levels, acute and chronic toxicity. Acute toxicity, especially hepatotoxicity, nephrotoxicity, and cardiotoxicity, could be determined during administration. Cardiotoxicity could be measured by left ventricular ejection fraction (LVEF), which directly shows the pumping ability of the heart (Florescu, Cinteza, & Vinereanu, 2013). For hepatotoxicity, clinical criteria of chemotherapy‐induced hepatotoxicity is regularly defined by the serum levels of alanine aminotransferase (ALT), aspartate aminotransferase (AST), total bilirubin (TBIL), alkaline phosphatase (ALP), and http://www.guidetopharmacology.org/GRAC/ObjectDisplayForward?objectId=1392 (γ‐GT) in which increases to 2 or 3 times higher than the normal upper limit, as acute liver injury is occurring (Y. C. Yu et al., 2017). Nephrotoxicity is defined by serum creatinine level and GFRs (measured by urine volume produced in particular time period) and has five stages from risk to end‐stage renal disease (ESRD; Horie et al., 2018). The criteria of biochemical examinations could guide oncologists and researchers to monitor possible toxicities, which can be used to determine the benefits of anticancer efficacy and, subsequently, to proceed or to cease treatment. In practice, a physician could take a more restricted posture towards advancement of ALT and AST levels based on normal ranges, to securely assure the ongoing therapy. Likewise, the above criteria could be applied to monitor the chronic toxicity in liver, heart, and kidney which potent natural compounds/conventional drugs combination could provide greater anticancer efficacy without exceeding about criteria.

Prospectively, the immunostimulatory effect of natural compounds in chemotherapy is a critical issue, as chemotherapy‐induced immunosuppression could cause severe opportunistic infections (Galluzzi, Buqué, Kepp, Zitvogel, & Kroemer, 2015). Some natural compounds and herbal products have proved as immunomodulators in vivo, such as Ashwagangha (Withania somnifera) and Brahmi (Bacopa monnieri), which improve https://www.guidetopharmacology.org/GRAC/LigandDisplayForward?ligandId=4968 and https://www.guidetopharmacology.org/GRAC/LigandDisplayForward?ligandId=4985 expression after LPS exposure (Yamada, Hung, Park, Park, & Lim, 2011). Moreover, the immunomodulating activity of astragaloside (the major components of huang‐qi, *Astragalus membranaceus*) has been linked to http://www.guidetopharmacology.org/GRAC/ObjectDisplayForward?objectId=1852 modulation that is essential for T‐cell activation (Qi, Gao, Hou, & Wan, 2017; Rheinlander, Schraven, & Bommhardt, 2018; Wan et al., 2013). Combining the immunostimulatory efficacy of herbal products with immunosuppressive chemotherapeutic drugs like gemcitabine, the side effects of immunosuppression might be relieved. Furthermore, these two studies propose a new approach for appraising the enhancement of the potential of natural compounds in combination with chemotherapeutic drugs. Before applying folk, herb, and natural compounds in combination therapy, the antitumor efficacy of folk, herb, and natural compounds needs to be assessed first.

## 4 CONCLUSION

**Figure 2:**
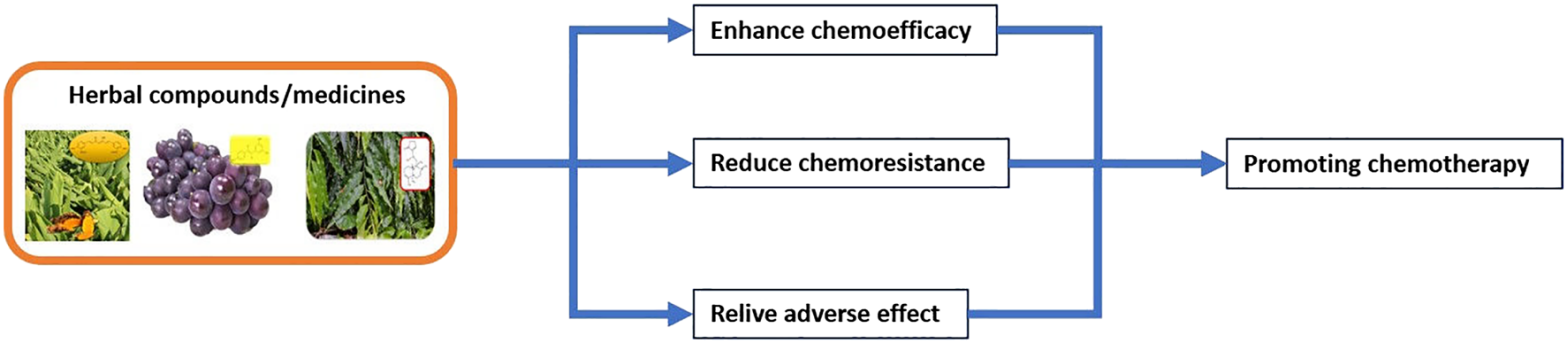
Putative mechanism of natural compounds in chemotherapeutic synergism

Collectively, the mechanisms of natural compounds acting as chemotherapeutic adjuvants could be summarized into three approaches: directly potentiating tumoricidal effect (sensitizing cancer cells to be more responsive to chemotherapeutic drugs), reversing chemoresistance (diminishing drug efflux or overcoming other mechanism to increase the accumulation of chemotherapeutic drugs in cancer cells), and alleviating toxicity induced by chemotherapeutic drugs (promoting the repairing mechanism in normal cells against damage of chemotherapeutic drugs; Figure 2). After demonstrating anticancer activity as monotherapy, natural compounds could further enhance their application by being chemotherapeutic adjuvants or cooperating drugs in combination therapy. Using TCM or traditional herbal medicine as a chemotherapeutic adjuvant in treating NSCLC or gastric cancer could improve the quality of life of patients, ameliorate myelosuppression, and possibly reduce mortality (Hou et al., 2017; Y. K. Lee, Bae, Yoo, & Cho, 2018; X. Wu et al., 2016). Further studies should look at herbal compounds or low MW compounds that can be applied as an alternative potent supplement for cancer therapy to attenuate any adverse effects and chemoresistance. However, the toxicity of herbal–drug interactions for liver or kidney injury needs to be extensively considered as a precaution during new drug discovery and development.

### 4.1 Nomenclature of targets and ligands

Key protein targets and ligands in this article are hyperlinked to corresponding entries in http://www.guidetopharmacology.org, the common portal for data from the UPHAR/BPS Guide to PHARMACOLOGY (Harding et al., 2018), and are permanently archived in the Concise Guide to PHARMACOLOGY 2017/18 (Alexander, Christopoulos et al., 2017; Alexander, Cidlowski et al., 2017; Alexander, Fabbro et al., 2017; Alexander, Kelly et al., 2017a, b).

## CONFLICT OF INTEREST

The authors declare no conflicts of interest.

## Supporting information

Data S1. Supporting Information

Data S2. Supporting Information
