# Supplementary material for: Natural compounds as potential adjuvants to cancer therapy: Preclinical evidence
Source: Br J Pharmacol. 2019 Nov 27;177(6):1409–23. doi: 10.1111/bph.14816 (PMC7056458; doi:10.1111/bph.14816)
Supplement: Supplementary file 1 — Data S1. Supporting Information [file BPH-177-1409-s001.pdf]

Supplementary T1 Reference list of Table 2

Adwas AA, Elkhoely AA, Kabel AM, Abdel-Rahman MN, & Eissa AA (2016). Anti-cancer and cardioprotective effects of indol-3-carbinol in doxorubicin-treated mice. J Infect Chemother 22**:** 36-43.

Al Sawah E, Marchion DC, Xiong Y, Ramirez IJ, Abbasi F, Boac BM, *et al.* (2015). The Chinese herb polyphyllin D sensitizes ovarian cancer cells to cisplatin-induced growth arrest. J Cancer Res Clin Oncol 141**:** 237-242.

Baharuddin P, Satar N, Fakiruddin KS, Zakaria N, Lim MN, Yusoff NM*, et al.* (2016). Curcumin improves the efficacy of cisplatin by targeting cancer stem-like cells through p21 and cyclin D1-mediated tumour cell inhibition in non-small cell lung cancer cell lines. Oncol Rep 35**:** 13-25.

Bahman AA, Abaza MSI, Khoushiash SI, & Al-Attiyah RJ (2018). Sequencedependent effect of sorafenib in combination with natural phenolic compounds on hepatic cancer cells and the possible mechanism of action. Int J Mol Med 42**:** 1695-1715.

Boridy S, Le PU, Petrecca K, & Maysinger D (2014). Celastrol targets proteostasis and acts synergistically with a heat-shock protein 90 inhibitor to kill human glioblastoma cells. Cell Death Dis 5**:** e1216.

Boueroy P, Hahnvajanawong C, Boonmars T, Saensa-ard S, Wattanawongdon W, Kongsanthia C*, et al.* (2017). Synergistic Effect of Forbesione From Garcinia hanburyi in Combination with 5-Fluorouracil on Cholangiocarcinoma. Asian Pac J Cancer Prev 18**:** 3343-3351.

Catanzaro D, Gabbia D, Cocetta V, Biagi M, Ragazzi E, Montopoli M*, et al.* (2018). Silybin counteracts doxorubicin resistance by inhibiting GLUT1 expression. Fitoterapia 124**:** 42-48.

Chen CF, Lu CC, Chiang JH, Chiu HY, Yang JS, Lee CY*, et al.* (2018). Synergistic inhibitory effects of cetuximab and curcumin on human cisplatin-resistant oral cancer CAR cells through intrinsic apoptotic process. Oncol Lett 16**:** 6323-6330.

Chen P, Li J, Jiang HG, Lan T, & Chen YC (2015). Curcumin reverses cisplatin resistance in cisplatin-resistant lung caner cells by inhibiting FA/BRCA pathway. Tumour Biol 36**:** 3591-3599.

Conti S, Vexler A, Edry-Botzer L, Kalich-Philosoph L, Corn BW, Shtraus N, *et al.* (2018). Combined acetyl-11-keto-beta-boswellic acid and radiation treatment inhibited glioblastoma tumor cells. PLoS One 13**:** e0198627.

D'Aguanno S, D'Agnano I, De Canio M, Rossi C, Bernardini S, Federici G*, et al.* (2012). Shotgun proteomics and network analysis of neuroblastoma cell lines treated with curcumin. Mol Biosyst 8**:** 1068-1077.

Di Y, De Silva F, Krol ES, & Alcorn J (2018). Flaxseed Lignans Enhance the Cytotoxicity of Chemotherapeutic Agents against Breast Cancer Cell Lines MDA-MB-231 and SKBR3. Nutr Cancer 70**:** 306-315.

Diaz-Chavez J, Fonseca-Sanchez MA, Arechaga-Ocampo E, Flores-Perez A, Palacios-Rodriguez Y, Dominguez-Gomez G, *et al.* (2013). Proteomic profiling reveals that resveratrol inhibits HSP27 expression and sensitizes breast cancer cells to doxorubicin therapy. PLoS One 8**:** e64378.

Dyshlovoy SA, Venz S, Shubina LK, Fedorov SN, Walther R, Jacobsen C*, et al.* (2014). Activity of aaptamine and two derivatives, demethyloxyaaptamine and isoaaptamine, in cisplatin-resistant germ cell cancer. J Proteomics 96**:** 223-239.

Elbaz HA, Lee I, Antwih DA, Liu J, Huttemann M, & Zielske SP (2014). Epicatechin stimulates mitochondrial activity and selectively sensitizes cancer cells to radiation. PLoS One 9**:** e88322.

Goder A, Nagel G, Kraus A, Dorsam B, Seiwert N, Kaina B, *et al.* (2015). Lipoic acid inhibits the DNA repair protein O 6-methylguanine-DNA methyltransferase (MGMT) and triggers its depletion in colorectal cancer cells with concomitant autophagy induction. Carcinogenesis 36**:** 817-831.

Guo Y, An H, Feng L, Liu Q, Wang S, & Zhang T (2014). Sinapine as an active compound for inhibiting the proliferation of Caco-2 cells via downregulation of P-glycoprotein. Food Chem Toxicol 67**:** 187-192.

Hahnvajanawong C, Wattanawongdon W, Chomvarin C, Anantachoke N, Kanthawong S, Sripa B, *et al.* (2014). Synergistic effects of isomorellin and forbesione with doxorubicin on apoptosis induction in human cholangiocarcinoma cell lines. Cancer Cell Int 14**:** 68.

Hanusova V, Caltova K, Svobodova H, Ambroz M, Skarka A, Murinova N, *et al.* (2017). The effects of beta-caryophyllene oxide and trans-nerolidol on the efficacy of doxorubicin in breast cancer cells and breast tumor-bearing mice. Biomed Pharmacother 95**:** 828-836.

Hao J, Li Z, Zhang C, Yu W, Tang Z, Li Y, *et al.* (2017). Targeting NF-kappaB/AP-2beta signaling to enhance antitumor activity of cisplatin by melatonin in hepatocellular carcinoma cells. Am J Cancer Res 7**:** 13-27.

Hintzpeter J, Seliger JM, Hofman J, Martin HJ, Wsol V, & Maser E (2016). Inhibition of human anthracycline reductases by emodin - A possible remedy for anthracycline resistance. Toxicol Appl Pharmacol 293**:** 21-29.

Ho TF, Peng YT, Chuang SM, Lin SC, Feng BL, Lu CH, *et al.* (2009). Prodigiosin down-regulates survivin to facilitate paclitaxel sensitization in human breast carcinoma cell lines. Toxicol Appl Pharmacol 235**:** 253-260.

Hu FW, Yu CC, Hsieh PL, Liao YW, Lu MY, & Chu PM (2017). Targeting oral cancer stemness and chemoresistance by isoliquiritigenin-mediated GRP78 regulation. Oncotarget 8**:** 93912-93923.

Huang Y, Wang K, Gu C, Yu G, Zhao D, Mai W, *et al.* (2018). Berberine, a natural plant alkaloid, synergistically sensitizes human liver cancer cells to sorafenib. Oncol Rep 40**:** 1525-1532.

Ito Y, Mitani T, Harada N, Isayama A, Tanimori S, Takenaka S, *et al.* (2013). Identification of carbonyl reductase 1 as a resveratrol-binding protein by affinity chromatography using 4'-amino-3,5-dihydroxy-trans-stilbene. J Nutr Sci Vitaminol (Tokyo) 59**:** 358-364.

Jiang G, Liu J, Ren B, Zhang L, Owusu L, Liu L, *et al.* (2017). Anti-tumor and chemosensitization effects of Cryptotanshinone extracted from Salvia miltiorrhiza Bge. on ovarian cancer cells in vitro. J Ethnopharmacol 205**:** 33-40.

Jin HR, Zhao J, Zhang Z, Liao Y, Wang CZ, Huang WH, *et al.* (2012). The antitumor natural compound falcarindiol promotes cancer cell death by inducing endoplasmic reticulum stress. Cell Death Dis 3**:** e376.

Joy B, Nishanth Kumar S, Soumya MS, Radhika AR, Vibin M, & Abraham A (2014). Embelin (2,5-dihydroxy-3-undecyl-p-benzoquinone): a bioactive molecule isolated from Embelia ribes as an effective photodynamic therapeutic candidate against tumor in vivo. Phytomedicine 21**:** 1292-1297.

Kang JH, Kang HS, Kim IK, Lee HY, Ha JH, Yeo CD, *et al.* (2015). Curcumin sensitizes human lung cancer cells to apoptosis and metastasis synergistically combined with carboplatin. Exp Biol Med (Maywood) 240**:** 1416-1425.

Kang Y, Hu W, Bai E, Zheng H, Liu Z, Wu J, *et al.* (2016). Curcumin sensitizes human gastric cancer cells to 5-fluorouracil through inhibition of the NFkappaB survival-signaling pathway. Onco Targets Ther 9**:** 7373-7384.

Lee SY, Rhee YH, Jeong SJ, Lee HJ, Lee HJ, Jung MH, *et al.* (2011). Hydrocinchonine, cinchonine, and quinidine potentiate paclitaxel-induced cytotoxicity and apoptosis via multidrug resistance reversal in MES-SA/DX5 uterine sarcoma cells. Environ Toxicol 26**:** 424-431.

Li Q, Zhan M, Chen W, Zhao B, Yang K, Yang J, *et al.* (2016). Phenylethyl isothiocyanate reverses cisplatin resistance in biliary tract cancer cells via glutathionylation-dependent degradation of Mcl-1. Oncotarget 7**:** 10271-10282.

Li QQ, Wang G, Liang H, Li JM, Huang F, Agarwal PK, *et al.* (2013). beta-Elemene promotes cisplatin-induced cell death in human bladder cancer and other carcinomas. Anticancer Res 33**:** 1421-1428.

Lim W, Park S, Bazer FW, & Song G (2017). Naringenin-Induced Apoptotic Cell Death in Prostate Cancer Cells Is Mediated via the PI3K/AKT and MAPK Signaling Pathways. J Cell Biochem 118**:** 1118-1131.

Lim W, Yang C, Bazer FW, & Song G (2016). Luteolin Inhibits Proliferation and Induces Apoptosis of Human Placental Choriocarcinoma Cells by Blocking the

PI3K/AKT Pathway and Regulating Sterol Regulatory Element Binding Protein Activity. Biol Reprod 95**:** 82.

Lin SR, & Weng CF (2018). PG-Priming Enhances Doxorubicin Influx to Trigger Necrotic and Autophagic Cell Death in Oral Squamous Cell Carcinoma. J Clin Med 7**:** pii: E375.

Liu J, Li M, Wang Y, & Luo J (2017). Curcumin sensitizes prostate cancer cells to radiation partly via epigenetic activation of miR-143 and miR-143 mediated autophagy inhibition. J Drug Target 25**:** 645-652.

Liu Q, Jiang H, Liu Z, Wang Y, Zhao M, Hao C, *et al.* (2011). Berberine radiosensitizes human esophageal cancer cells by downregulating homologous recombination repair protein RAD51. PLoS One 6**:** e23427.

Liu Q, Sun Y, Zheng JM, Yan XL, Chen HM, Chen JK, *et al.* (2015). Formononetin sensitizes glioma cells to doxorubicin through preventing EMT via inhibition of histone deacetylase 5. Int J Clin Exp Pathol 8**:** 6434-6441.

Liu X, Duan C, Ji J, Zhang T, Yuan X, Zhang Y, *et al.* (2017). Cucurbitacin B induces autophagy and apoptosis by suppressing CIP2A/PP2A/mTORC1 signaling axis in human cisplatin resistant gastric cancer cells. Oncol Rep 38**:** 271-278.

Liu Z, Ma L, Wen ZS, Cheng YX, & Zhou GB (2014). Ethoxysanguinarine Induces Inhibitory Effects and Downregulates CIP2A in Lung Cancer Cells. ACS Med Chem Lett 5**:** 113-118.

Lou W, Chen Y, Zhu KY, Deng H, Wu T, & Wang J (2017). Polyphyllin I Overcomes EMT-Associated Resistance to Erlotinib in Lung Cancer Cells via IL-6/STAT3 Pathway Inhibition. Biol Pharm Bull 40**:** 1306-1313.

Louisa M, Soediro TM, & Suyatna FD (2014). In vitro modulation of P-glycoprotein, MRP-1 and BCRP expression by mangiferin in doxorubicin-treated MCF-7 cells. Asian Pac J Cancer Prev 15**:** 1639-1642.

Lu Z, Lai ZQ, Leung AWN, Leung PS, Li ZS, & Lin ZX (2017). Exploring brusatol as a new anti-pancreatic cancer adjuvant: biological evaluation and mechanistic studies. Oncotarget 8**:** 84974-84985.

Ma L, Li W, Wang R, Nan Y, Wang Q, Liu W, *et al.* (2015). Resveratrol enhanced anticancer effects of cisplatin on non-small cell lung cancer cell lines by inducing mitochondrial dysfunction and cell apoptosis. Int J Oncol 47**:** 1460-1468.

Ma L, Wang R, Nan Y, Li W, Wang Q, & Jin F (2016). Phloretin exhibits an anticancer effect and enhances the anticancer ability of cisplatin on non-small cell lung cancer cell lines by regulating expression of apoptotic pathways and matrix metalloproteinases. Int J Oncol 48**:** 843-853.

Manouchehri JM, Turner KA, & Kalafatis M (2018). TRAIL-Induced Apoptosis in TRAIL-Resistant Breast Carcinoma Through Quercetin Cotreatment. Breast Cancer (Auckl) 12**:** 1178223417749855.

Matsui Y, Watanabe J, Ding S, Nishizawa K, Kajita Y, Ichioka K, *et al.* (2010). Dicoumarol enhances doxorubicin-induced cytotoxicity in p53 wild-type urothelial cancer cells through p38 activation. BJU Int 105**:** 558-564.

Meng X, Dong X, Wang W, Yang L, Zhang X, Li Y, *et al.* (2018). Natural Borneol Enhances Paclitaxel-Induced Apoptosis of ESCC Cells by Inactivation of the PI3K/AKT. J Food Sci 83**:** 1436-1443.

Milczarek M, Mielczarek L, Lubelska K, Dabrowska A, Chilmonczyk Z, Matosiuk D, *et al.* (2018). In Vitro Evaluation of Sulforaphane and a Natural Analog as Potent Inducers of 5-Fluorouracil Anticancer Activity. Molecules 23.

Mundhe NA, Kumar P, Ahmed S, Jamdade V, Mundhe S, & Lahkar M (2015). Nordihydroguaiaretic acid ameliorates cisplatin induced nephrotoxicity and potentiates its anti-tumor activity in DMBA induced breast cancer in female Sprague-Dawley rats. Int Immunopharmacol 28**:** 634-642.

Namazi Sarvestani N, Sepehri H, Delphi L, & Moridi Farimani M (2018). Eupatorin and Salvigenin Potentiate Doxorubicin-Induced Apoptosis and Cell Cycle Arrest in HT-29 and SW948 Human Colon Cancer Cells. Asian Pac J Cancer Prev 19**:** 131-139.

Park S, Cho DH, Andera L, Suh N, & Kim I (2013). Curcumin enhances TRAIL-induced apoptosis of breast cancer cells by regulating apoptosis-related proteins. Mol Cell Biochem 383**:** 39-48.

Pastorek M, Simko V, Takacova M, Barathova M, Bartosova M, Hunakova L*, et al.* (2015). Sulforaphane reduces molecular response to hypoxia in ovarian tumor cells independently of their resistance to chemotherapy. Int J Oncol 47**:** 51-60.

Patel S, Waghela B, Shah K, Vaidya F, Mirza S, Patel S*, et al.* (2018). Silibinin, A Natural Blend In Polytherapy Formulation For Targeting Cd44v6 Expressing Colon Cancer Stem Cells. Sci Rep 8**:** 16985.

Pham MQ, Iscache AL, Pham QL, & Gairin JE (2016). Cytotoxic, apoptotic, and sensitization properties of ent-kaurane-type diterpenoids from Croton tonkinensis Gagnep on human liver cancer HepG2 and Hep3b cell lines. Fundam Clin Pharmacol 30**:** 137-146.

Piska K, Koczurkiewicz P, Wnuk D, Karnas E, Bucki A, Wojcik-Pszczola K*, et al.* (2019). Synergistic anticancer activity of doxorubicin and piperlongumine on DU-145 prostate cancer cells - The involvement of carbonyl reductase 1 inhibition. Chem Biol Interact 300**:** 40-48.

Poornima P, Kumar VB, Weng CF, & Padma VV (2014). Doxorubicin induced apoptosis was potentiated by neferine in human lung adenocarcima, A549 cells. Food Chem Toxicol 68**:** 87-98.

Qian J, Xia M, Liu W, Li L, Yang J, Mei Y*, et al.* (2019). Glabridin resensitizes p-glycoprotein-overexpressing multidrug-resistant cancer cells to conventional chemotherapeutic agents. Eur J Pharmacol 852**:** 231-243.

Richards CE, Vellanki SH, Smith YE, & Hopkins AM (2018). Diterpenoid natural compound C4 (Crassin) exerts cytostatic effects on triple-negative breast cancer cells via a pathway involving reactive oxygen species. Cell Oncol (Dordr) 41**:** 35-46.

Saikia M, Retnakumari AP, Anwar S, Anto NP, Mittal R, Shah S*, et al.* (2018). Heteronemin, a marine natural product, sensitizes acute myeloid leukemia cells towards cytarabine chemotherapy by regulating farnesylation of Ras. Oncotarget 9**:** 18115-18127.

Sanchez BG, Bort A, Mateos-Gomez PA, Rodriguez-Henche N, & Diaz-Laviada I (2019). Combination of the natural product capsaicin and docetaxel synergistically kills human prostate cancer cells through the metabolic regulator AMP-activated kinase. Cancer Cell Int 19**:** 54.

Santos GC, Almeida MR, Antunes L, & Bianchi M (2016). Effect of bixin on DNA damage and cell death induced by doxorubicin in HL60 cell line. Hum Exp Toxicol 35**:** 1319-1327.

Shi DB, Li XX, Zheng HT, Li DW, Cai GX, Peng JJ, *et al.* (2014). Icariin-mediated inhibition of NF-kappaB activity enhances the in vitro and in vivo antitumour effect of 5-fluorouracil in colorectal cancer. Cell Biochem Biophys 69**:** 523-530.

Srijiwangsa P, Ponnikorn S, & Na-Bangchang K (2018). Effect of beta-Eudesmol on NQO1 suppression-enhanced sensitivity of cholangiocarcinoma cells to chemotherapeutic agents. BMC Pharmacol Toxicol 19**:** 32.

Su S, Cheng X, & Wink M (2015). Natural lignans from Arctium lappa modulate P-glycoprotein efflux function in multidrug resistant cancer cells. Phytomedicine 22**:** 301-307.

Su W, Huang L, Ao Q, Zhang Q, Tian X, Fang Y, *et al.* (2011). Noscapine sensitizes chemoresistant ovarian cancer cells to cisplatin through inhibition of HIF-1alpha. Cancer Lett 305**:** 94-99.

Sun H, Huang M, Yao N, Hu J, Li Y, Chen L, *et al.* (2017). The cycloartane triterpenoid ADCX impairs autophagic degradation through Akt overactivation and promotes apoptotic cell death in multidrug-resistant HepG2/ADM cells. Biochem Pharmacol 146**:** 87-100.

Sun J, Yeung CA, Co NN, Tsang TY, Yau E, Luo K, *et al.* (2012). Clitocine reversal of P-glycoprotein associated multi-drug resistance through down-regulation of transcription factor NF-kappaB in R-HepG2 cell line. PLoS One 7**:** e40720.

Tang N, Zhang J, & Du Y (2010). [Curcumin promoted the apoptosis of cisplain-resistant human lung carcinoma cells A549/DDP through down-regulating miR-186*]. Zhongguo Fei Ai Za Zhi 13**:** 301-306.

Taylor-Harding B, Agadjanian H, Nassanian H, Kwon S, Guo X, Miller C, *et al.* (2012). Indole-3-carbinol synergistically sensitises ovarian cancer cells to bortezomib treatment. Br J Cancer 106**:** 333-343.

Tian M, Tian D, Qiao X, Li J, & Zhang L (2019). Modulation of Myb-induced NF-kB -STAT3 signaling and resulting cisplatin resistance in ovarian cancer by dietary factors. J Cell Physiol.

Tormo JR, Royo I, Gallardo T, Zafra-Polo MC, Hernandez P, Cortes D, *et al.* (2003). In vitro antitumor structure-activity relationships of threo/trans/threo mono-tetrahydrofuranic acetogenins: correlations with their inhibition of mitochondrial complex I. Oncol Res 14**:** 147-154.

Tseng HS, Wang YF, Tzeng YM, Chen DR, Liao YF, Chiu HY, *et al.* (2017). Aloe-Emodin Enhances Tamoxifen Cytotoxicity by Suppressing Ras/ERK and PI3K/mTOR in Breast Cancer Cells. Am J Chin Med 45**:** 337-350.

Tyszka-Czochara M, Konieczny P, & Majka M (2017). Caffeic Acid Expands Anti-Tumor Effect of Metformin in Human Metastatic Cervical Carcinoma HTB-34 Cells: Implications of AMPK Activation and Impairment of Fatty Acids De Novo Biosynthesis. Int J Mol Sci 18**:** 462.

Tyszka-Czochara M, Lasota M, & Majka M (2018). Caffeic Acid and Metformin Inhibit Invasive Phenotype Induced by TGF-beta1 in C-4I and HTB-35/SiHa Human Cervical Squamous Carcinoma Cells by Acting on Different Molecular Targets. Int J Mol Sci 19**:** 266.

Venier NA, Colquhoun AJ, Sasaki H, Kiss A, Sugar L, Adomat H, *et al.* (2015). Capsaicin: a novel radio-sensitizing agent for prostate cancer. Prostate 75**:** 113-125.

Villar VH, Vogler O, Barcelo F, Gomez-Florit M, Martinez-Serra J, Obrador-Hevia A, *et al.* (2014). Oleanolic and maslinic acid sensitize soft tissue sarcoma cells to doxorubicin by inhibiting the multidrug resistance protein MRP-1, but not P-glycoprotein. J Nutr Biochem 25**:** 429-438.

Wang GZ, Liu YQ, Cheng X, & Zhou GB (2015). Celastrol induces proteasomal degradation of FANCD2 to sensitize lung cancer cells to DNA crosslinking agents. Cancer Sci 106**:** 902-908.

Wang HC, Lee AY, Chou WC, Wu CC, Tseng CN, Liu KY, *et al.* (2012). Inhibition of ATR-dependent signaling by protoapigenone and its derivative sensitizes cancer cells to interstrand cross-link-generating agents in vitro and in vivo. Mol Cancer Ther 11**:** 1443-1453.

Wang J, & Yuan Z (2013). Gambogic acid sensitizes ovarian cancer cells to doxorubicin through ROS-mediated apoptosis. Cell Biochem Biophys 67**:** 199-206.

Wang X, Deng R, Lu Y, Xu Q, Yan M, Ye D, *et al.* (2013). Gambogic acid as a non-competitive inhibitor of ATP-binding cassette transporter B1 reverses the multidrug resistance of human epithelial cancers by promoting ATP-binding cassette transporter B1 protein degradation. Basic Clin Pharmacol Toxicol 112**:** 25-33.

Wang Y, Lu HL, Liu YD, Yang LY, Jiang QK, Zhu XJ, *et al.* (2017). Cryptotanshinone sensitizes antitumor effect of paclitaxel on tongue squamous cell carcinoma growth by inhibiting the JAK/STAT3 signaling pathway. Biomed Pharmacother 95**:** 1388-1396.

Wang YT, Liu HS, & Su CL (2014). Curcumin-enhanced chemosensitivity of FDA-approved platinum (II)-based anti-cancer drugs involves downregulation of nuclear endonuclease G and NF-kappaB as well as induction of apoptosis and G2/M arrest. Int J Food Sci Nutr 65**:** 368-374.

Wei W, Liu C, Qin D, Song L, Xia L, Lei H, *et al.* (2016). Targeting peroxiredoxin I potentiates 1,25-dihydroxyvitamin D3-induced cell differentiation in leukemia cells. Mol Med Rep 13**:** 2201-2207.

Xie B, Lu YY, Luo ZH, Qu Z, Zheng CG, Huang XA, *et al.* (2019). Tenacigenin B ester derivatives from Marsdenia tenacissima actively inhibited CYP3A4 and enhanced in vivo antitumor activity of paclitaxel. J Ethnopharmacol 235**:** 309-319.

Xin M, Wang Y, Ren Q, & Guo Y (2019). Formononetin and metformin act synergistically to inhibit growth of MCF-7 breast cancer cells in vitro. Biomed Pharmacother 109**:** 2084-2089.

Xu D, Tian W, & Shen H (2013). P-gp upregulation may be blocked by natural curcuminoids, a novel class of chemoresistance-preventing agent. Mol Med Rep 7**:** 115-121.

Xu XM, Zhang Y, Qu D, Liu HB, Gu X, Jiao GY, *et al.* (2013). Combined anticancer activity of osthole and cisplatin in NCI-H460 lung cancer cells in vitro. Exp Ther Med 5**:** 707-710.

Xu Z, Chen L, Xiao Z, Zhu Y, Jiang H, Jin Y, *et al.* (2018). Potentiation of the anticancer effect of doxorubicinin drug-resistant gastric cancer cells by tanshinone IIA. Phytomedicine 51**:** 58-67.

Yang ES, Choi MJ, Kim JH, Choi KS, & Kwon TK (2011). Combination of withaferin A and X-ray irradiation enhances apoptosis in U937 cells. Toxicol In Vitro 25**:** 1803-1810.

Yang YI, Lee KT, Park HJ, Kim TJ, Choi YS, Shih Ie M, *et al.* (2012). Tectorigenin sensitizes paclitaxel-resistant human ovarian cancer cells through downregulation of the Akt and NFkappaB pathway. Carcinogenesis 33**:** 2488-2498.

Yao K, Jiang X, He L, Tang Y, Yin G, Zeng Q, *et al.* (2015). Anacardic acid sensitizes prostate cancer cells to radiation therapy by regulating H2AX expression. Int J Clin Exp Pathol 8**:** 15926-15932.

Yu LL, Wu JG, Dai N, Yu HG, & Si JM (2011). Curcumin reverses chemoresistance of human gastric cancer cells by downregulating the NF-kappaB transcription factor. Oncol Rep 26**:** 1197-1203.

Zhang H, Ozaki I, Hamajima H, Iwane S, Takahashi H, Kawaguchi Y, *et al.* (2011). Vitamin K2 augments 5-fluorouracil-induced growth inhibition of human hepatocellular carcinoma cells by inhibiting NF-kappaB activation. Oncol Rep 25**:** 159-166.

Zhang J, Liu J, Xu X, & Li L (2017). Curcumin suppresses cisplatin resistance development partly via modulating extracellular vesicle-mediated transfer of MEG3 and miR-214 in ovarian cancer. Cancer Chemother Pharmacol 79**:** 479-487.

Zhang S, Sagawa K, Arnold RD, Tseng E, Wang X, & Morris ME (2010). Interactions between the flavonoid biochanin A and P-glycoprotein substrates in rats: in vitro and in vivo. J Pharm Sci 99**:** 430-441.

Zhao Q, Guan J, Qin Y, Ren P, Zhang Z, Lv J, *et al.* (2018). Curcumin sensitizes lymphoma cells to DNA damage agents through regulating Rad51-dependent homologous recombination. Biomed Pharmacother 97**:** 115-119.

Zhao X, Fang Y, Yang Y, Qin Y, Wu P, Wang T, *et al.* (2015). Elaiophylin, a novel autophagy inhibitor, exerts antitumor activity as a single agent in ovarian cancer cells. Autophagy 11**:** 1849-1863.

Zhong Y, Zhang F, Sun Z, Zhou W, Li ZY, You QD, *et al.* (2013). Drug resistance associates with activation of Nrf2 in MCF-7/DOX cells, and wogonin reverses it by down-regulating Nrf2-mediated cellular defense response. Mol Carcinog 52**:** 824-834.

Zong L, Cheng G, Liu S, Pi Z, Liu Z, & Song F (2019). Reversal of multidrug resistance in breast cancer cells by a combination of ursolic acid with doxorubicin. J Pharm Biomed Anal 165**:** 268-275.

Zou J, Zhu L, Jiang X, Wang Y, Wang Y, Wang X, *et al.* (2018). Curcumin increases breast cancer cell sensitivity to cisplatin by decreasing FEN1 expression. Oncotarget 9**:** 11268-11278.

Zou L, Wang D, Hu Y, Fu C, Li W, Dai L, *et al.* (2017). Drug resistance reversal in ovarian cancer cells of paclitaxel and borneol combination therapy mediated by PEG-PAMAM nanoparticles. Oncotarget 8**:** 60453-60468.

Zou M, Xu C, Li H, Zhang X, & Fan W (2018). 3,3'-Diindolylmethane suppresses ovarian cancer cell viability and metastasis and enhances chemotherapy sensitivity via STAT3 and Akt signaling in vitro and in vivo. Arch Biochem Biophys.
