# Supplementary material for: Natural compounds as potential adjuvants to cancer therapy: Preclinical evidence
Source: Br J Pharmacol. 2019 Nov 27;177(6):1409–23. doi: 10.1111/bph.14816 (PMC7056458; doi:10.1111/bph.14816)
Supplement: Supplementary file 2 — Data S2. Supporting Information [file BPH-177-1409-s002.pdf]

Supplementary T2 Reference list in Table 3

Hershman DL, Unger JM, Crew KD, Minasian LM, Awad D, Moinpour CM, *et al.* (2013). Randomized double-blind placebo-controlled trial of acetyl-L-carnitine for the prevention of taxane-induced neuropathy in women undergoing adjuvant breast cancer therapy. J Clin Oncol 31**:** 2627-2633.

Hosseinzadeh L, Behravan J, Mosaffa F, Bahrami G, Bahrami A, & Karimi G (2011). Curcumin potentiates doxorubicin-induced apoptosis in H9c2 cardiac muscle cells through generation of reactive oxygen species. Food Chem Toxicol 49**:** 1102-1109.

Melchardt T, Magnes T, Weiss L, Grundbichler M, Strasser M, Hufnagl C, *et al.* (2014). Liver toxicity during temozolomide chemotherapy caused by Chinese herbs. BMC Complement Altern Med 14**:** 115.

Saleh EM, El-awady RA, Eissa NA, & Abdel-Rahman WM (2012). Antagonism between curcumin and the topoisomerase II inhibitor etoposide: a study of DNA damage, cell cycle regulation and death pathways. Cancer Biol Ther 13**:** 1058-1071.

Strippoli S, Lorusso V, Albano A, & Guida M (2013). Herbal-drug interaction induced rhabdomyolysis in a liposarcoma patient receiving trabectedin. BMC Complement Altern Med 13**:** 199.
